# Supplementary material for: New Antifungal Compound: Impact of Cosolvency, Micellization and Complexation on Solubility and Permeability Processes
Source: Pharmaceutics. 2021 Nov 4;13(11):1865. doi: 10.3390/pharmaceutics13111865 (PMC8621413; doi:10.3390/pharmaceutics13111865)
Supplement: Supplementary file 1 [file pharmaceutics-13-01865-s001.zip › pharmaceutics-1421436-supplementary.pdf]

pharmaceutics

Supplementary Material

New Antifungal Compound: Impact of Cosolvency, Micellization and Complexation on Solubility and Permeability Processes

Tatyana V. Volkova, Olga R. Simonova and German L. Perlovich *

Gennady Alekseevich Krestov Institute of Solution Chemistry of the Russian Academy of Sciences , 153045 Ivanovo, Russia; vtv@isc-ras.ru (T.V.V.); ors@isc-ras.ru (O.R.S.)
* Correspondence: glp@isc-ras.ru

Citation: Volkova, T.V.; Simonova, O.R.; Perlovich, G.L. New Antifungal Compound: Impact of Cosolvency, Micellization and Complexation on Solubility and Permeability Processes. *Pharmaceutics* **2021**, *13*, 1865. https://doi.org/10.3390/pharmaceutics13111865

Academic Editors: Anton V. Syroeshkin and Elena V. Uspenskaya

Received: 29 September 2021
Accepted: 1 November 2021
Published: 4 November 2021

Publisher's Note: MDPI stays neutral with regard to jurisdictional claims in published maps and institutional affiliations.

Copyright: © 2021 by the authors. Licensee MDPI, Basel, Switzerland. This article is an open access article distributed under the terms and conditions of the Creative Commons Attribution (CC BY) license (http://creativecommons.org/licenses/by/4.0/).

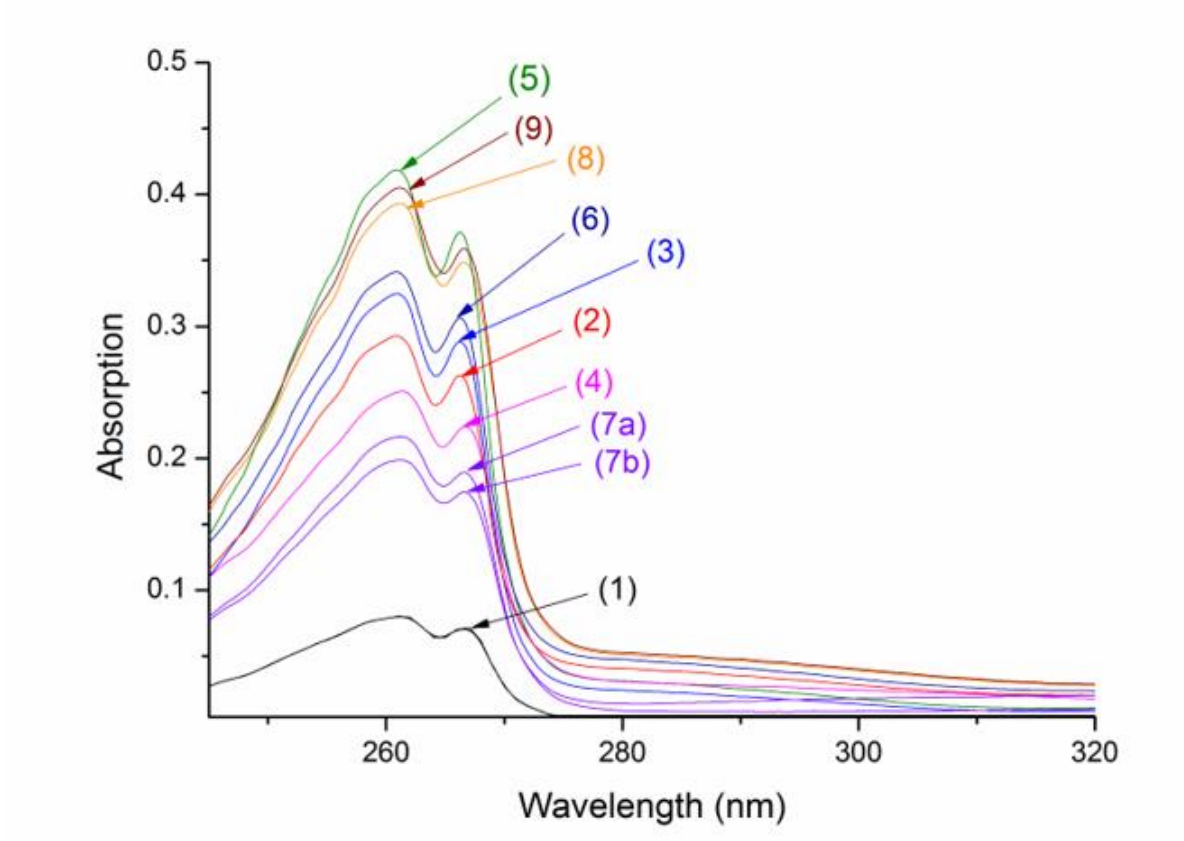

Figure S1. Absorption spectra of S-119 in buffer solution (pH 7.4) and in the presence of different excipients: (1) - pure buffer; (2) - PEG; (3) - PVP; (4) - Brij S20; (5) - F-127; (6) - α-CD; (7a) - β-CD 0.001M; (7b) - β-CD 0.004 M; (8) - HP-β-CD; (9) O-M-β-CD.

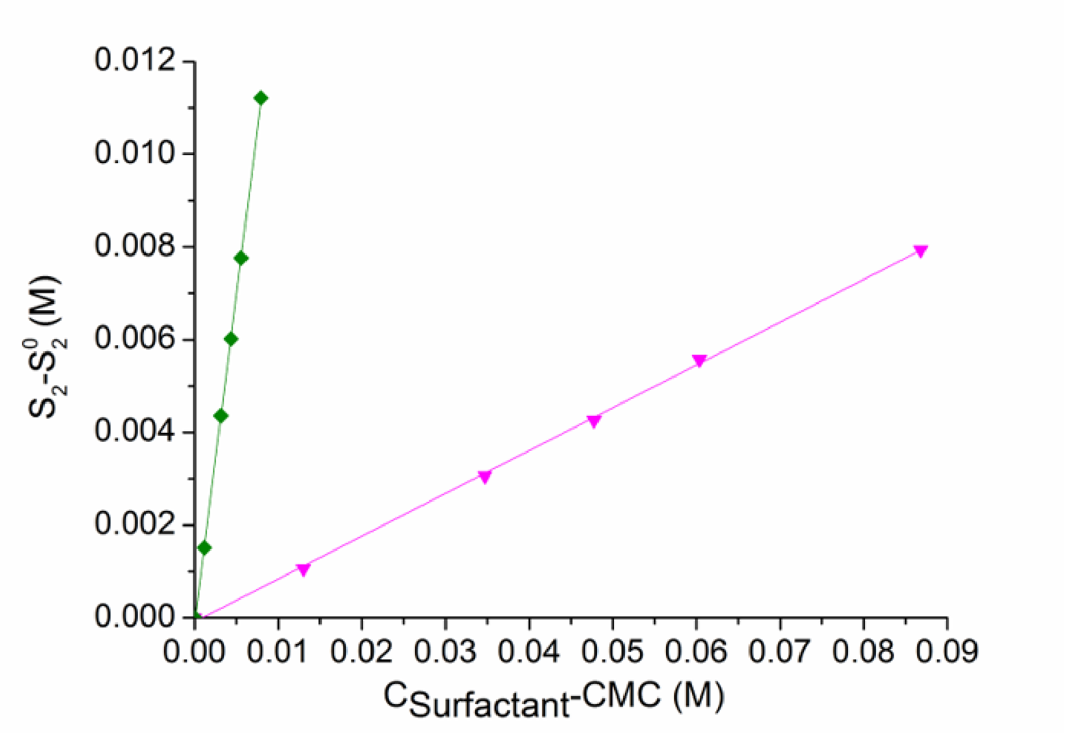

Figure S2. Plots correlating the S-119 solubility (S−S^0) in Brij S20 (▼) and F127 (◆) solutions at pH 7.4 on surfactant concentration ($C_{surfactant}$−CMC) at 25 °C.

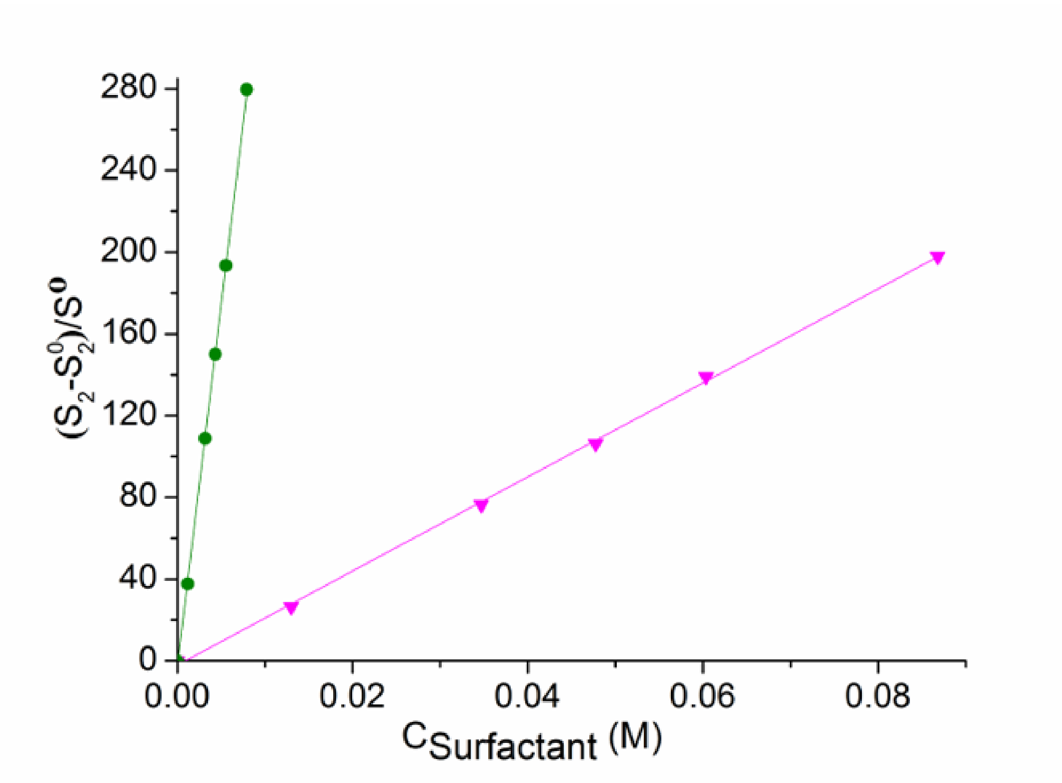

Figure S3. Plots correlating the solubility in Brij S20 (▼) and F127 (◆) at pH 7.4 normalized by the aqueous solubility (S_2 / S_2^0) at pH 7.4 on surfactant concentration ($C_{Surfactant}$) at 25 °C.

Table S1. Solubility of S-119 in different media at 25.0±0.1ºC.

Medium	Solubility, M	
	Buffer pH 2.0	Buffer pH 7.4
Pure buffer	$(4.45 \pm 0.14)\cdot 10^{-3}$	$(4.01 \pm 0.11)\cdot 10^{-5}$
PEG 6000 (1.5 *w/v*%)	$(4.74 \pm 0.11)\cdot 10^{-3}$	$(7.82 \pm 0.15)\cdot 10^{-5}$
PEG 6000 (4 *w/v*%)	$(5.24 \pm 0.15)\cdot 10^{-3}$	$(8.41 \pm 0.16)\cdot 10^{-4}$
PEG 6000 (5.5 *w/v*%)	$(5.57 \pm 0.09)\cdot 10^{-3}$	$(4.14 \pm 0.07)\cdot 10^{-3}$
PEG 6000 (7 *w/v*%)	$(5.86 \pm 0.11)\cdot 10^{-3}$	$(6.80 \pm 0.08)\cdot 10^{-3}$
PEG 6000 (10 *w/v*%)	$(6.47 \pm 0.17)\cdot 10^{-3}$	$(7.73 \pm 0.21)\cdot 10^{-3}$
PEG 35000 (1.5 *w/v*%)	$(4.59 \pm 0.14)\cdot 10^{-3}$	$(9.01 \pm 0.26)\cdot 10^{-5}$
PEG 35000 (4 *w/v*%)	$(4.80 \pm 0.08)\cdot 10^{-3}$	$(4.28 \pm 0.14)\cdot 10^{-4}$
PEG 35000 (5.5 *w/v*%)	$(4.94 \pm 0.13)\cdot 10^{-3}$	$(1.53 \pm 0.03)\cdot 10^{-3}$
PEG 35000 (7 *w/v*%)	$(5.05 \pm 0.09)\cdot 10^{-3}$	$(5.59 \pm 0.14)\cdot 10^{-3}$
PEG 35000 (10 *w/v*%)	$(5.32 \pm 0.14)\cdot 10^{-3}$	$(7.16 \pm 0.16)\cdot 10^{-3}$
PVP (1.5 *w/v*%)	$(4.60 \pm 0.05)\cdot 10^{-3}$	$(2.81 \pm 0.04)\cdot 10^{-4}$
PVP (4 *w/v*%)	$(5.03 \pm 0.08)\cdot 10^{-3}$	$(2.93 \pm 0.04)\cdot 10^{-4}$
PVP (5.5 *w/v*%)	$(5.16 \pm 0.14)\cdot 10^{-3}$	$(1.25 \pm 0.03)\cdot 10^{-3}$
PVP (7 *w/v*%)	$(5.45 \pm 0.14)\cdot 10^{-3}$	$(7.56 \pm 0.24)\cdot 10^{-3}$
PVP (10 *w/v*%)	$(5.86 \pm 0.14)\cdot 10^{-3}$	$(9.29 \pm 0.22)\cdot 10^{-3}$
Brij S20 (1.5 *w/v*%)	$(3.89 \pm 0.03)\cdot 10^{-3}$	$(1.10 \pm 0.04)\cdot 10^{-3}$
Brij S20 (4 *w/v*%)	$(3.48 \pm 0.14)\cdot 10^{-3}$	$(3.10 \pm 0.04)\cdot 10^{-3}$
Brij S20 (5.5 *w/v*%)	$(3.45 \pm 0.05)\cdot 10^{-3}$	$(4.30 \pm 0.08)\cdot 10^{-3}$
Brij S20 (7 *w/v*%)	$(3.45 \pm 0.07)\cdot 10^{-3}$	$(5.62 \pm 0.08)\cdot 10^{-3}$
Brij S20 (10 *w/v*%)	$(3.45 \pm 0.07)\cdot 10^{-3}$	$(7.98 \pm 0.14)\cdot 10^{-3}$
Pluronic F-127 (1.5 *w/v*%)	$(4.45 \pm 0.11)\cdot 10^{-3}$	$(1.55 \pm 0.03)\cdot 10^{-3}$
Pluronic F-127 (4 *w/v*%)	$(4.43 \pm 0.13)\cdot 10^{-3}$	$(4.40 \pm 0.06)\cdot 10^{-3}$
Pluronic F-127 (5.5 *w/v*%)	$(4.14 \pm 0.07)\cdot 10^{-3}$	$(6.06 \pm 0.16)\cdot 10^{-3}$
Pluronic F-127 (7 *w/v*%)	$(3.99 \pm 0.04)\cdot 10^{-3}$	$(7.80 \pm 0.15)\cdot 10^{-3}$
Pluronic F-127 (10 *w/v*%)	-	$(1.13 \pm 0.11)\cdot 10^{-2}$

α-CD (1.5 *w/v*%)	$(4.80 \pm 0.14)\cdot 10^{-3}$	$(2.15 \pm 0.06)\cdot 10^{-4}$
α-CD (4 *w/v*%)	$(5.33 \pm 0.18)\cdot 10^{-3}$	$(5.40 \pm 0.07)\cdot 10^{-4}$
α-CD (5.5 *w/v*%)	$(5.69 \pm 0.08)\cdot 10^{-3}$	$(7.12 \pm 0.10)\cdot 10^{-4}$
α-CD (7 *w/v*%)	$(6.03 \pm 0.17)\cdot 10^{-3}$	$(9.06 \pm 0.11)\cdot 10^{-4}$
β-CD (0.11 *w/v*%)	$(4.56 \pm 0.04)\cdot 10^{-3}$	$(1.97 \pm 0.02)\cdot 10^{-4}$
β-CD (0.17 *w/v*%)	$(4.62 \pm 0.12)\cdot 10^{-3}$	$(2.71 \pm 0.06)\cdot 10^{-4}$
β-CD (0.23 *w/v*%)	$(4.67 \pm 0.11)\cdot 10^{-3}$	$(3.50 \pm 0.05)\cdot 10^{-4}$
β-CD (0.34 *w/v*%)	$(4.78 \pm 0.09)\cdot 10^{-3}$	$(4.96 \pm 0.14)\cdot 10^{-4}$
β-CD (0.45 *w/v*%)	$(4.58 \pm 0.04)\cdot 10^{-3}$	$(2.04 \pm 0.10)\cdot 10^{-4}$
HP-β-CD (1.5 *w/v*%)	$(5.09 \pm 0.16)\cdot 10^{-3}$	$(9.66 \pm 0.24)\cdot 10^{-4}$
HP-β-CD (4 *w/v*%)	$(6.11 \pm 0.17)\cdot 10^{-3}$	$(2.41 \pm 0.05)\cdot 10^{-3}$
HP-β-CD (7 *w/v*%)	$(7.42 \pm 0.10)\cdot 10^{-3}$	$(4.26 \pm 0.14)\cdot 10^{-3}$
HP-β-CD (10 *w/v*%)	$(8.71 \pm 0.10)\cdot 10^{-3}$	$(6.07 \pm 0.12)\cdot 10^{-3}$
O-M-β-CD (1.5 *w/v*%)	$(5.21 \pm 0.11)\cdot 10^{-3}$	$(1.38 \pm 0.02)\cdot 10^{-3}$
O-M-β-CD (4 *w/v*%)	$(6.56 \pm 0.15)\cdot 10^{-3}$	$(3.55 \pm 0.06)\cdot 10^{-3}$
O-M-β-CD (5.5 *w/v*%)	$(7.33 \pm 0.14)\cdot 10^{-3}$	$(4.87 \pm 0.11)\cdot 10^{-3}$
O-M-β-CD (7 *w/v*%)	$(8.13 \pm 0.19)\cdot 10^{-3}$	$(6.26 \pm 0.15)\cdot 10^{-3}$

Table S2. Stability constants (K_S) and complexation efficiency (CE) of S-119 complexes with cyclodextrins in buffer solutions at pH 2.0 and pH 7.4 at 25.0±0.1 °C.

	**K_S*, M^{-1}**		**CE** (%)**		**Molar Ratio*** (drug:CD)**	
	**pH 2.0**	**pH 7.4**	**pH 2.0**	**pH 7.4**	**pH 2.0**	**pH 7.4**
S-119/α-CD	5.0 ± 0.2	304.4 ± 17	2.2	1.2	1:45	1:83
S-119/β-CD	27.4 ± 1.0	4424.9± 162.7	12.2	17.7	1:2	1:2
S-119/HP-β-CD	14.0 ± 0.6	2261.0 ± 79.0	6.2	9.1	1:17	1:28
S-119/O-M-β-CD	18.7 ± 0.7	3693.4 ± 185.3	8.3	14.8	1:13	1:7

$$^{*}K_S = \frac{\text{slope}}{S_2^0(1-\text{slope})}\,;\; ^{**}CE = \frac{\text{slope}}{(1-\text{slope})}\times 100\%\,;\; ^{***}\text{Drug}:\text{CD} = 1:\left(1+\frac{1}{CE}\right)$$

Table S3. Gibbs free energy of solubilization process (ΔG_S^0), micelle formation ($\Delta G_{m/w}^0$) and complexation (ΔG_C^0) for S-119 in buffer pH 2.0 and pH 7.4 at different excipients concentration (C_{exc}) at 25 °C.

***C_{exc}* (*w/v*%)**	**ΔG_S^0 (kJ·mol^{-1})**	**ΔG_S^0 (kJ·mol^{-1})**	**$\Delta G_{m/w}^0$ (kJ·mol^{-1})**	**$\Delta G_{m/w}^0$ (kJ·mol^{-1})**	**ΔG_C^0 (kJ·mol^{-1})**	**ΔG_C^0 (kJ·mol^{-1})**
	pH 2.0	pH 7.4	pH 2.0	pH 7.4	pH 2.0	pH 7.4
			PEG 6000			
1.5	−0.16	−1.66				
4.0	−0.41	−7.54				
5.5	−0.56	−11.49				
7.0	−0.68	−12.72				
10.0	−0.93	−13.04				
			PEG 35000			
1.5	−0.08	−2.01				
4.0	−0.19	−5.87				
5.5	−0.26	−9.03				
7.0	−0.31	−12.24				
10.0	−0.44	−12.85				
			PVP			
1.5	−0.08	−4.83				
4.0	−0.30	−4.93				
5.5	−0.37	−8.53				
7.0	−0.50	−12.99				
10.0	−0.68	−13.50				
			Brij S20			
1.5	0.33	−8.21				
4.0	0.61	−10.78				
5.5	0.63	−11.59	-	−19.19	-	-
7.0	0.63	−12.25				
10.0	0.63	−13.12				
			F127			
1.5	0	−9.06				
4.0	0.01	−11.65				
5.5	0.18	−12.44	-	−25.96	-	-
7.0	0.27	−13.06				
10.0	-	−13.97				
			α-CD			
1.5	−0.19	−4.16				
4.0	−0.45	−6.45	-	-	−4.00	−14.17
5.5	−0.61	−7.13				
7.0	−0.75	−7.73				
			β-CD			
0.11	−0.06	−3.94	-	-		−20.81
0.17	−0.09	−4.74			−8.21	

0.23	−0.12	−5.37				
0.34	−0.18	−6.23				
0.45	−0.07	−4.03				
HP-β-CD						
1.5	−0.33	−7.89	-	-	−6.55	−19.15
4.0	−0.79	−10.16				
7.0	−1.27	−11.57				
10.0	−1.66	−12.44				
O-M-β-CD						
1.5	−0.39	−8.77	-	-	−7.26	−20.36
4.0	−0.96	−11.11				
5.5	−1.24	−11.90				
7.0	−1.49	−12.52				
